# Marked Changes in Serum Amyloid A Distribution and High-Density Lipoprotein Structure during Acute Inflammation

**DOI:** 10.1155/2021/9241259

**Published:** 2021-01-27

**Authors:** Shitsuko Shimano, Ryunosuke Ohkawa, Mayu Nambu, Mai Sasaoka, Azusa Yamazaki, Yuki Fujii, Yuna Horiuchi, Shao-Jui Lai, Takahiro Kameda, Naoya Ichimura, Koji Fujita, Shuji Tohda, Minoru Tozuka

**Affiliations:** ^1^Clinical Laboratory, Medical Hospital, Tokyo Medical and Dental University (TMDU), 1-5-45 Yushima, Bunkyo-ku, Tokyo 113-8519, Japan; ^2^Analytical Laboratory Chemistry, Graduate School of Medical and Dental Sciences, Tokyo Medical and Dental University (TMDU), 1-5-45 Yushima, Bunkyo-ku, Tokyo 113-8510, Japan; ^3^Orthopaedic and Spinal Surgery, Medical Hospital, Tokyo Medical and Dental University (TMDU), 1-5-45 Yushima, Bunkyo-ku, Tokyo 113-8519, Japan; ^4^Life Science Research Center, Nagano Children's Hospital, 3100 Toyoshina, Azumino 399-8288, Japan

## Abstract

High-density lipoprotein- (HDL-) cholesterol measurements are generally used in the diagnosis of cardiovascular diseases. However, HDL is a complicated heterogeneous lipoprotein, and furthermore, it can be converted into dysfunctional forms during pathological conditions including inflammation. Therefore, qualitative analysis of pathophysiologically diversified HDL forms is important. A recent study demonstrated that serum amyloid A (SAA) can remodel HDL and induce atherosclerosis not only over long periods of time, such as during chronic inflammation, but also over shorter periods. However, few studies have investigated rapid HDL remodeling. In this study, we analyzed HDL samples from patients undergoing orthopedic surgery inducing acute inflammation. We enrolled 13 otherwise healthy patients who underwent orthopedic surgery. Plasma samples were obtained on preoperative day and postoperative days (POD) 1-7. SAA, apolipoprotein A-I (apoA-I), and apolipoprotein A-II (apoA-II) levels in the isolated HDL were determined. HDL particle size, surface charge, and SAA and apoA-I distributions were also analyzed. In every patient, plasma SAA levels peaked on POD3. Consistently, the HDL apoA-I : apoA-II ratio markedly decreased at this timepoint. Native-polyacrylamide gel electrophoresis and high-performance liquid chromatography revealed the loss of small HDL particles during acute inflammation. Furthermore, HDL had a decreased negative surface charge on POD3 compared to the other timepoints. All changes observed were SAA-dependent. SAA-dependent rapid changes in HDL size and surface charge were observed after orthopedic surgery. These changes might affect the atheroprotective functions of HDL, and its analysis can be available for the qualitative HDL assessment.

## 1. Introduction

High-density lipoprotein (HDL) is a well-known, multifunctional particle that has been shown to suppress the progression of atherosclerosis by numerous epidemiological and experimental studies [1–4]. HDL and other lipoproteins transport lipids through the lymphatic and circulatory systems; many studies provided insights into the causal relationship between lipids and atherogenesis [5]. Consequently, HDL-cholesterol (HDL-C) measurements are generally used in the diagnosis of cardiovascular diseases, and many attempts have been made to pharmaceutically increase its levels [6–10]. However, HDL is a complicated and heterogeneous lipoprotein, and furthermore, it can be converted into dysfunctional forms during pathological conditions like diabetes [11, 12], oxidative stress [13, 14], and inflammation [15, 16]. Therefore, qualitative analysis of pathophysiologically diversified HDL forms is important and has been widely conducted.

Chronic inflammation induces constant HDL remodeling that can lead to a higher risk of acute coronary syndrome. For example, levels of small HDL particles were low in patients with rheumatoid arthritis, who also had elevated coronary calcification [17]. Patients infected with human immunodeficiency virus also display increased large HDL particles and decreased small HDL particles [18].

One of the most important HDL remodeling factors is serum amyloid A (SAA). Previous studies reported that during inflammation, the blood SAA level increased up to 579-3560 *μ*g/mL, more than 1,000 times its basal level, following its production in the liver [19–21]. The majority of the SAA produced binds to HDL and displaces its main component, apolipoprotein A-I (apoA-I) [22–25].

Strikingly, a recent study showed that mice receiving a single injection of an adenoviral vector encoding human SAA1 displayed increased atherosclerosis, despite only brief elevations in SAA levels [26]. This suggests that SAA can have acute effects on HDL, in addition to the long-term effects observed during chronic inflammation. However, few studies have analyzed SAA distribution changes in the HDL of human subjects. In this study, we report rapid, SAA-dependent changes in HDL characteristics in patients who underwent orthopedic surgery.

## 2. Materials and Methods

### 2.1. Samples

Patients samples in this study were the residuals of blood samples obtained for laboratory analyses at the Clinical Laboratory of the Medical Hospital at the Tokyo Medical and Dental University. Whole blood samples were submitted to the laboratory before (PRE) and just after (POST) orthopedic surgery and on postoperative days (PODs) 1, 3, and 6 or 7 between April 2017 and March 2018. Blood samples from patients who had been diagnosed with any other medical disease (e.g., liver disease, diabetes, and other inflammatory diseases) were excluded. After anonymizing the patient samples, the untraceable blood tubes collected in vacuum tubes containing ethylenediaminetetraacetic acid dipotassium (Terumo, Tokyo, Japan) were centrifuged at 4°C and 2,150 × *g* for 30 min for plasma collection. The samples were stored at -80°C and analyzed within 4 months. Human experiments complied with all relevant national regulations and institutional policies and were performed in accordance with the tenets of the Declaration of Helsinki. The study design was disclosed publicly to patients which were offered the opportunity to opt out of this project for the use of the residual samples. The study was approved by the institutional research ethics committee of the Faculty of Medicine, Tokyo Medical and Dental University (M2016-049).

### 2.2. HDL Isolation

HDL (*d* = 1.063–1.210 g/mL) was isolated from patient plasma samples by ultracentrifugation as previously described [27]. The isolated HDL fraction was dialyzed against phosphate-buffered saline (PBS), stored at 4°C, and used within 3 weeks.

### 2.3. SAA and Lipid Measurements

SAA concentrations in plasma and HDL fractions were determined using a commercially available latex agglutination-turbidimetric immunoassay kit (LZ SAA, Eiken Chemical, Tokyo, Japan). Plasma albumin and HDL-C were measured using L-Type Wako ALB-BCP and MetaboLead HDL-C kits, respectively (FUJIFILM Wako Pure Chemical Corporation, Osaka, Japan, and Kyowa Medex, Tokyo, Japan). These measurements were performed using a LABOSPECT 008 automatic analyzer (Hitachi High Technologies, Tokyo, Japan). Protein levels in HDL fractions were measured by Lowry et al.'s method [28].

### 2.4. Electrophoresis and Western Blot Analysis

HDL samples were resolved by sodium dodecyl sulfate- (SDS-) polyacrylamide gel (PAGE) on 16% gels under nonreducing conditions, and by Native-PAGE on 8% gels followed by Coomassie Brilliant Blue (CBB) staining and western blot analysis, respectively, as previously described [23]. apoA-I was detected using a goat anti-apoA-I polyclonal antibody (1 : 1,000 dilution, Academy Bio-Medical Company, Houston, TX, USA) and horseradish peroxidase- (HRP-) conjugated rabbit anti-goat IgG (1 : 2,000 dilution, Medical & Biological Laboratories, Aichi, Japan), and SAA was detected using a rabbit anti-human SAA polyclonal antibody (1 : 1,200 dilution, ASSAYPRO，MO, USA) and HRP-conjugated goat anti-rabbit IgG (1 : 50,000 dilution, Beckman Coulter, CA, United States). apoA-I and SAA were visualized with 3,3′-diaminobenzidine-4HCl and H_2_O_2_ or with ECL Prime Western Blotting Detection Reagent (GE Healthcare, New York, USA). Agarose electrophoresis was performed using TITAN GEL (Helena, Saitama, Japan) according to the manufacturer's protocol, and the resolved lipoproteins were stained with 0.1% Fat Red 7B. Semiquantification and analysis of the relative mobility of each band were performed by densitometry on a CS Analyzer 4 (ATTO, Tokyo, Japan).

### 2.5. High-Performance Liquid Chromatography (HPLC)

To investigate changes in HDL particle size, patient HDL fractions were analyzed on an HPLC system equipped with a LC-20ADVP pump, a DGU-20A degassing unit, a CTO-20A column oven, an SPD-20A ultraviolet detector, and an SIL-20AC autoinjector (Shimadzu Corporation, Kyoto, Japan). Each sample (40 *μ*L) was injected into serially connected size exclusion columns (PROTEIN KW-803 and KW-804; 300 mm × 8.0 mm i.d., Shodex, Tokyo, Japan), eluted with PBS at a flow rate of 1 mL/min, and monitored by absorbance at 280 nm.

### 2.6. Statistical Analysis

All data represent the mean ± standard deviation (SD) unless otherwise stated. Statistical significance was assessed using SPSS version 20.0 (IBM, Chicago, IL, USA) by Spearman's rank correlation coefficient test or unpaired Student's *t*-test. *p* < 0.05 was considered statistically significant.

## 3. Results

### 3.1. Changes in SAA Levels before and after Surgery

**Figure 1:**
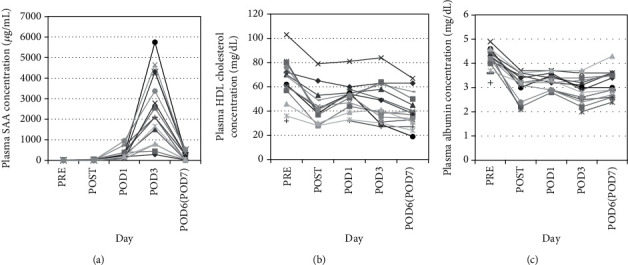
Changes in plasma SAA and HDL-C levels after surgery. Plasma samples were collected from patients (*n* = 13) before (PRE) and just after (POST) orthopedic surgery and on postoperative days (POD) 1, 3, and 6 or 7. Plasma SAA (a), HDL-C (b), and albumin (c) levels were determined.

SAA concentrations in patient plasma samples (*n* = 13) were determined at the PRE, POST, and POD1, 3, and 6/7 timepoints. All patients except for case 2 (27.7 *μ*g/mL) had low plasma SAA levels (mean ± SD: 5.1 ± 2.7 *μ*g/mL) near the reference range (<8 *μ*g/mL) PRE, and these values generally remained low (3.7 ± 2.0 *μ*g/mL) POST, although cases 3 and 12 displayed slight increases in SAA (22.5 and 53.7 *μ*g/mL, respectively; Figure 1(a)). Conversely, plasma SAA levels in all patients increased markedly by POD1 (318.1 ± 265.9 *μ*g/mL) and POD3 (2552.2 ± 1805.8 *μ*g/mL) and then decreased (211.9 ± 186.1 *μ*g/mL) by POD6/7. In each patient, plasma SAA peaked on POD3; however, the extent of the increase varied. Plasma HDL-C and albumin levels were also measured and displayed similar trends, decreasing by 34 ± 14% for HDL-C and 28 ± 12% for albumin POST compared to PRE, then remaining largely unchanged through POD3. At POD6/7, HDL-C and albumin levels decreased and increased, respectively (Figures 1(b) and 1(c)).

### 3.2. Changes in the HDL Apolipoprotein Ratio

**Figure 2:**
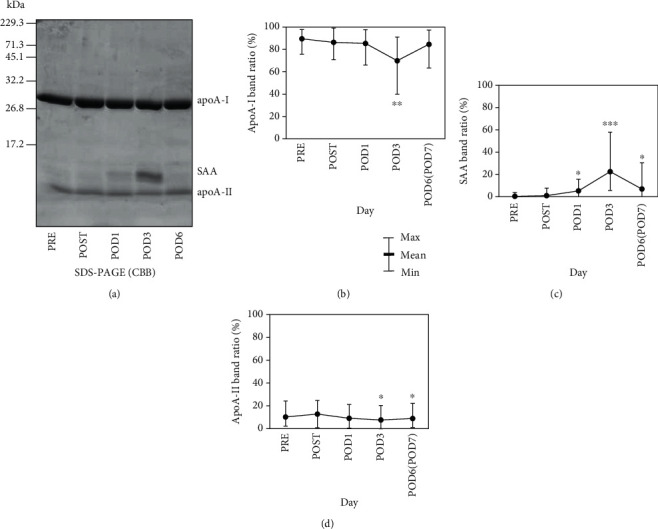
Changes in HDL apolipoprotein levels after surgery. HDL fractions were isolated from patient plasma (*n* = 13) before (PRE) and just after (POST) orthopedic surgery and on postoperative days (POD) 1, 3, and 6 or 7 by ultracentrifugation. Fractions were resolved by SDS-PAGE (4 *μ*g/lane) followed by CBB staining. A representative profile is shown (a). Bands at 28, 12, and 8 kDa represent apoA-I, SAA, and apoA-II, respectively. Band intensities were semiquantified by densitometry. The intensities for apoA-I (b), SAA (c), and apoA-II (d) are shown as percentages of the summed intensity for all three bands. Values represent the mean, min, and max. ^∗^*p* < 0.05, ^∗∗^*p* < 0.005, and ^∗∗∗^*p* < 0.001 vs. POST by paired *t*-test.

Each HDL sample was analyzed by SDS-PAGE followed by CBB staining (Figure 2(a)). Two bands at apparent molecular masses of 28 and 8 kDa, corresponding to apoA-I and apoA-II monomers, respectively, were clearly observed in every HDL fraction at all timepoints. Bands of various intensities were also observed at 12 kDa, corresponding to SAA. Consistent with the plasma SAA level, the SAA band appeared on POD1, peaked on POD3, and was absent on POD6 (Figure 2(a)). To investigate relative changes in apolipoprotein levels, the sum of the intensities of the apoA-I, apoA-II, and SAA bands was calculated at each timepoint and used to determine the intensity percentages of the individual bands. The relative intensity of the apoA-I band was significantly decreased (by 16.5%; *p* = 0.013) on POD3 compared to POST and had recovered by POD6/7. Conversely, the relative amount of SAA was significantly increased (by 21.6%; *p* < 0.001) on POD3 and returned to its basal level by POD6/7 (Figures 2(b) and 2(c)). However, the relative intensity changes for apoA-II did not resemble those for apoA-I. A slight decrease of 5.1% (*p* = 0.01) was observed on POD3, which remained on POD6/7 (Figure 2(d)).

### 3.3. Effect of Increased SAA on HDL Particle Size

**Figure 3:**
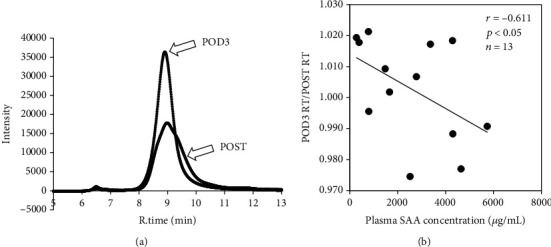
Association between HDL particle size and plasma SAA levels. HDL fractions from patients (*n* = 13) just after (POST) orthopedic surgery and on postoperative day 3 (POD3) were isolated by ultracentrifugation, and 40 *μ*L of 0.5 mg/mL HDL was resolved by HPLC. A representative HPLC profile is shown (a). The ratio of the retention times on POD3 and POST was compared to the plasma SAA concentration on POD3 (b). The correlation was estimated by Spearman's rank correlation coefficient test.

As we observed high amounts of SAA bound to HDL, we next examined changes in HDL particle size by HPLC. When the HPLC profiles of HDL fractions POST and on POD3 were compared, the retention time was slightly shorter on POD3, and the peak was sharper (Figure 3(a)). To investigate the association between the shortened retention time and the plasma SAA level, the ratio of the retention time on POD3 to POST was compared with the plasma SAA level on POD3. The ratio was significantly negatively correlated with the SAA level (*r* = −0.611, *p* < 0.05, *n* = 13; Figure 3(b)).

### 3.4. Changes in HDL apoA-I Distribution

**Figure 4:**
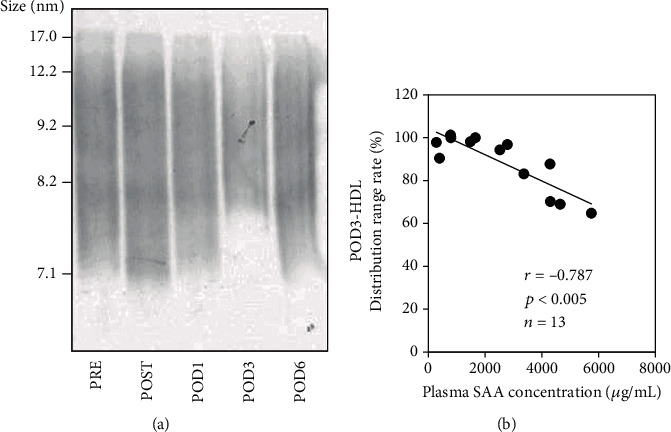
Changes in HDL apoA-I distribution after surgery. HDL fractions were isolated from patient plasma (*n* = 13) before (PRE) and just after (POST) orthopedic surgery and on postoperative days (POD) 1, 3, and 6 or 7 by ultracentrifugation. Fractions were subjected to Native-PAGE (1.0 *μ*g/lane) followed by western blotting for apoA-I. A representative profile is shown (a). For each patient, the HDL particle size range was determined by densitometry and the percentage of range decrease on POD3 compared to POST was compared with the plasma SAA level on POD3 (b). The correlation was estimated by Spearman's rank correlation coefficient test.

Patient HDL fractions were resolved by Native-PAGE followed by western blotting for apoA-I. In PRE HDL samples, apoA-I ranged from 7.1 to 17.0 nm in size (Figure 4(a)). Smaller HDL particles were absent on POD3 but present on POD6/7. The percentage of decrease in HDL particle size range from POST to POD3 was compared with the POD3 plasma SAA level, and the values were significantly negatively correlated (*r* = −0.787, *p* < 0.005, *n* = 13; Figure 4(b)).

### 3.5. Changes in HDL SAA Distribution

**Figure 5:**
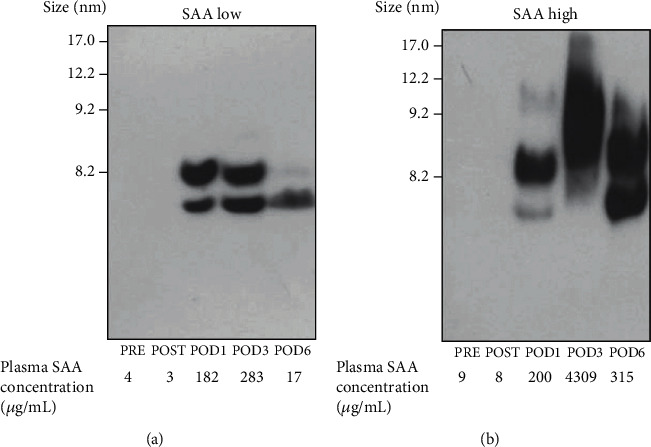
Changes in HDL SAA distribution after surgery. HDL fractions were isolated from patient plasma (*n* = 13) before (PRE) and just after (POST) orthopedic surgery and on postoperative days (POD) 1, 3, and 6 or 7 by ultracentrifugation. Fractions were subjected to Native-PAGE followed by western blotting for SAA. Each sample was adjusted to 500 *μ*g/lane SAA before loading. In cases with lower SAA concentrations, the maximum volume was loaded. Representative HDL profiles from patients with low (a) and high (b) plasma SAA values are shown.

Since we observed HDL particle size changes by HPLC and Native-PAGE analyses, we next examined the SAA distribution in HDL by western blot analysis.  Figure 5 shows representative HDL profiles of patients with relatively low and high SAA levels on POD3 (Figures 5(a) and 5(b), respectively). After adjusting SAA levels to 500 *μ*g/lane, each HDL sample was resolved by Native-PAGE. In patients with low SAA, two large, noticeable bands were observed at particle sizes of 6.2 and 7.8 nm on POD1 and POD3 (Figure 5(a)). Conversely, in patients with high SAA, SAA was distributed on larger HDL particles on POD3, and the range of SAA distribution was wider compared to low SAA samples (Figure 5(b)).

### 3.6. Changes in HDL Surface Charges

**Figure 6:**
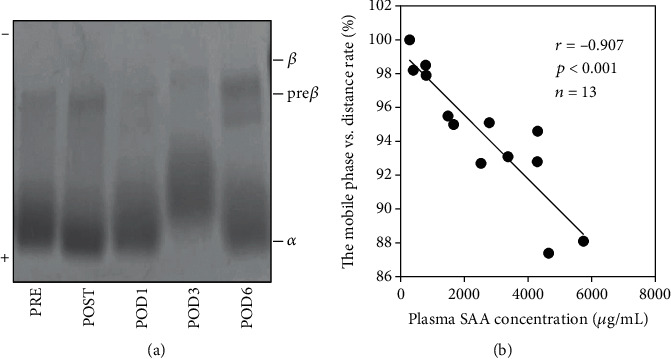
Changes in HDL surface charge after surgery. HDL fractions were isolated from patient plasma (*n* = 13) before (PRE) and just after (POST) orthopedic surgery and on postoperative days (POD) 1, 3, and 6 or 7 by ultracentrifugation. Fractions were analyzed by agarose gel electrophoresis followed by Fat Red 7B staining (HDL, 0.5 *μ*g/lane). A representative HDL profile is shown (a). The distance of HDL migration was measured by densitometry. The percentage by which migration decreased from POST to POD3 was compared with the plasma SAA level on POD3 (b). The correlation was estimated by Spearman's rank correlation coefficient test.

The surface charges of the HDL samples were analyzed by agarose gel electrophoresis. HDL particles were observed in the alpha fraction PRE and POST; however, the relative mobility of the HDL bands was shorter on POD3 than at other timepoints (Figure 6(a)). When the distance migrated by HDL was compared with the SAA level on POD3, a significant negative correlation was observed (*r* = −0.907, *p* < 0.001, *n* = 13; Figure 6(b)).

## 4. Discussion

A few studies have demonstrated rapid HDL remodeling due to increased SAA in human subjects. Zimetti et al. compared the HDL of 59 subjects with acute-phase reaction (APR) related to infections, oncological causes, or autoimmune diseases and control subjects without APR and reported that in patients with APR, apoA-I-containing and medium-sized HDL particles were reduced, and HDL function was impaired [29]. Jahangiri et al. reported similar reductions in HDL-C and apoA-I levels, as well as decreased cholesteryl ester transfer protein levels in the HDL of patients after cardiac surgery compared to before surgery [30]. However, the heterogeneity of HDL particles observed even in healthy subjects makes it difficult to determine how SAA affects HDL structure. In addition, preexisting conditions such as coronary artery disease can make analyzing these effects even more complicated, even when comparing HDL from the same patient at different timepoints. Therefore, in this study, we analyzed HDL samples from patients undergoing orthopedic surgery who had no additional major diseases or medical issues.

SAA levels in all patients showed notable increases 3 days after surgery and decreased rapidly over the next 4 days, similar to changes reported in patients with acute inflammation [31]. In addition, HDL-C levels decreased just after surgery, before SAA levels increased. Since albumin displayed the same trend as HDL-C, these decreases were thought to be due to intravenous rehydration. Therefore, to investigate the effects of increased SAA on HDL, we compared HDL characteristics on POD1, 3, and 6/7 with the HDL POST, not PRE. Displacement of HDL apoA-I by SAA has been shown both *in vitro* and *in vivo* [22–24]. Consistently, reduced relative apoA-I levels were observed on POD3, with a reciprocal change observed in SAA. Conversely, only slight changes in apoA-II levels were observed. These results are consistent with previous studies on patients with severe diseases, including septicemia, septic abortion, and bacterial dysentery [22] and indicate that apoA-I release from HDL due to competition from SAA occurs *in vivo*.

To investigate the association between the extent of SAA binding and HDL remodeling, we analyzed changes in HDL particle size, apoA-I and SAA distributions, and surface charge. In HPLC analysis, the shorter retention time of the peak top suggested a shift toward larger HDL particles. As equal amounts of HDL were injected to the HPLC, the marked change of the sharpness of the peak also indicated a narrowed HDL particle size range. These results were consistent with the apoA-I distribution by Native-PAGE. HDL size remodeling in these patients depended on their plasma SAA levels, consistent with previous reports [22, 32]. Since large changes in HDL-C concentrations were not observed between PRE and POD3, the reduction in small HDL particles was likely not due to catabolism, but rather SAA-induced HDL enlargement. Consistent with this, SAA was mainly distributed on larger HDL particles. However, a previous study of lipopolysaccharide administration to *SAA* knockout mice reported paradoxical results to ours, suggesting that the HDL size increase was associated with increased surface phospholipid content, not increased SAA [33]. In their Native-PAGE profile, the HDL size range, even in wild-type mice, was quite different from ours. Although additional phospholipid analysis would be instructive, this contradiction could also be simply due to differences in the organisms and inflammation induction methods used.

In our previous study, the surface charge of HDL differed between samples with low and high SAA [23]. However, these HDL samples were obtained from patients with different backgrounds (e.g., primary disease and inflammation condition), and we were unable to control for these differences. In this study, we analyzed otherwise healthy patients undergoing orthopedic surgery and observed positive changes in HDL surface charge that were SAA-dependent.

## 5. Conclusions

Our study reveals rapid apoA-I displacement, size remodeling, and surface charge changes in HDL that correspond to fluctuations in SAA levels in the same individuals at different timepoints. Recent studies from our group and others have demonstrated that SAA affects HDL functions, including its antioxidant ability [23, 34] and cholesterol efflux capacity [30, 35, 36]. Therefore, analysis of the SAA-specific HDL remodeling can be available for the qualitative HDL assessment as a biomarker.

## Data Availability

The data used to support the findings of this study are available from the corresponding author (Ryunosuke Ohkawa, Graduate School of Medical and Dental Sciences, Tokyo Medical and Dental University (TMDU), ohkawa.alc@tmd.ac.jp) upon request.
